# Adapting Lot Quality Assurance Sampling to accommodate imperfect diagnostic tests: application to COVID-19 serosurveillance in Haiti

**DOI:** 10.1186/s12889-022-14206-5

**Published:** 2022-11-29

**Authors:** Isabel R. Fulcher, Mary Clisbee, Wesler Lambert, Fernet Renand Leandre, Bethany Hedt-Gauthier

**Affiliations:** 1 Department of Global Health and Social Medicine, Harvard Medical School, 641 Huntington Ave, Boston, MA USA; 2 Department of Research, Zanmi Lasante, Santo 18A, Croix-des-Bouquets, Haïti; 3 Department of Research, Education and Strategic Information, Santo 18A, Croix-des-Bouquets, Haïti; 4 Division of Global Health Equity, Brigham and Women’s Hospital, 800 Boylston Street Suite 300, Boston, USA; 5 Department of Biostatistics, Harvard T.H. Chan School of Public Health, 677 Huntington Ave, Boston, MA USA

**Keywords:** Lot Quality Assurance Sampling, Serosurveys, Diagnostic testing, COVID-19

## Abstract

**Background:**

Lot Quality Assurance Sampling (LQAS), a tool used for monitoring health indicators in low resource settings resulting in “high” or “low” classifications, assumes that determination of the trait of interest is perfect. This is often not true for diagnostic tests, with imperfect sensitivity and specificity. Here, we develop Lot Quality Assurance Sampling for Imperfect Tests (LQAS-IMP) to address this issue and apply it to a COVID-19 serosurveillance study design in Haiti.

**Methods:**

We first derive a modified procedure, LQAS-IMP, that accounts for the sensitivity and specificity of a diagnostic test to yield correct classification errors. We then apply the novel LQAS-IMP to design an LQAS system to classify prevalence of SARS-CoV-2 antibodies among healthcare workers at eleven Zanmia Lasante health facilities in Haiti. Finally, we show the performance of the LQAS-IMP procedure in a simulation study.

**Results:**

We found that when an imperfect diagnostic test is used, the classification errors in the standard LQAS procedure are larger than specified. In the modified LQAS-IMP procedure, classification errors are consistent with the specified maximum classification error. We then utilized the LQAS-IMP procedure to define valid systems for sampling at eleven hospitals in Haiti.

**Conclusion:**

The LQAS-IMP procedure accounts for imperfect sensitivity and specificity in system design; if the accuracy of a test is known, the use of LQAS-IMP extends LQAS to applications for indicators that are based on laboratory tests, such as SARS-CoV-2 antibodies.

**Supplementary Information:**

The online version contains supplementary material available at 10.1186/s12889-022-14206-5.

## Background

As the world responds to the ongoing COVID-19 pandemic, timely and accurate surveillance is imperative to monitor the spread of the virus. SARS-CoV-2 virologic and serologic testing capacity is essential for individual clinical care, contact tracing and containment strategies, and monitoring for population-level dynamics. However, testing capacity in low- and middle-income countries (LMICs) remains hindered by limited laboratory infrastructure and technicians, inability to compete on the global market to procure tests, absence of quality controls capability for the procured tests reagents, and limited resources to purchase the necessary tests [1]. Therefore, any testing activity in LMICs must be highly efficient, maximizing the information gained while minimizing the number of tests used.

Lot Quality Assurance Sampling (LQAS) is a classification procedure used to categorize an area as “high” or “low” on some trait of interest [2, 3]. LQAS is used widely in monitoring and evaluation of health indicators in low resource settings [4, 5] because the method produces results that can be quickly linked to program response, can be implemented without need for advanced statistical training or software, and because it often requires smaller sample sizes than traditional estimation procedures. An LQAS system is defined by a sample size and decision rule for classification, determined by user-specified definitions of high and low thresholds for performance and limits to the amount of classification errors tolerated at these thresholds.

A key assumption underlying the design of LQAS systems is that, for any individual sample, determination of the trait of interest is perfect. This is an unrealistic assumption in many settings, particularly for diagnostic tests where the occurrence of false positives and negatives is ubiquitous and often a known quantity. If the accuracy of the diagnostic test is not taken into account, the LQAS system can be deeply flawed resulting in sample sizes and decision rules that have higher classification errors than that specified by the user in the design. While methods that account for diagnostic testing accuracy have been well established for prevalence estimation [6, 7], there is currently no method to account for imperfect diagnostic tests in the design of LQAS systems.

In this paper, we propose a new method for determining an LQAS system that accounts for the sensitivity and specificity of a diagnostic test. We present simulation studies to demonstrate the utility of our method and the impact of inaccurate estimates of sensitivity and specificity in various settings. We discuss the design of an LQAS system specific to the use of SARS-CoV-2 antibody tests among health care workers in Haiti as a motivating example for this method.

## Methods

### Overview of traditional LQAS Systems

In its most traditional form, the sample size, *n*, and decision rule, *d*, are determined by the population size, *N*, and then four parameters defined by users based on the specific context and goals. The four parameters are:

- ${p}_{u}$, the upper prevalence threshold by which an area is classified as high;
- ${p}_{l}$, the lower prevalence threshold by which an area is classified as low;
- $\alpha$, the probability that a high area is mistakenly classified as low; and
- $\beta$, the probability that a low area is mistakenly classified as high;

The sample size and decision rule are determined by finding the minimum *n* and corresponding *d*, such that the following are true:

1 $$\begin{gathered} \alpha >P\left( {X<d|{p_u},n,N} \right) \\ =\sum\nolimits_{{i=0}}^{{d - 1}} {P\left( {X=i|{p_u},n,N} \right)} \\ \end{gathered}$$

2 $$\begin{gathered} \beta >P\left( {X\underline {>} \;d|{p_l},n,N} \right) \\ =\sum\nolimits_{{i=d}}^{n} {P\left( {X=i|{p_l},n,N} \right)} \\ \end{gathered}$$

where *X* is a random variable denoting the number of persons with the trait of interest. Equation (1) states that the probability of sampling *n* individuals and observing fewer than *d* individuals with the trait of interest if the prevalence is ${p}_{u}$and thereby erroneously classifying as “low” is less than $\alpha$. Equation (2) states that the probability of sampling *n* individuals and observing *d* or more individuals with the trait of interest if the prevalence is ${p}_{l}$ and thereby erroneously classifying as “high” is less than $\beta$.

If working with small population sizes, for example facilities with a small number of health care workers, it is advantageous to take into account the population size, *N*, in these calculations as smaller population sizes will require a lower sample size. For small population sizes, the above probabilities are calculated using the hypergeometric distribution. That is, $X\sim HGM\left(N,n,{p}_{i}\right),$where *i* corresponds to the lower or upper prevalence threshold. If you have a sufficiently large population, then *N* is not necessary and the above probabilities can be calculated using the binomial distribution for *X*. There are freely available online tools and software packages to calculate the LQAS system based on both scenarios [8, 9].

### Accounting for imperfect testing properties

A problem with the traditional approach is that it assumes the method used to identify a “case” is perfect. That is, all positives are true positives and all negatives are true negatives. However, the majority of diagnostic tests suffer from imperfect diagnostic capabilities characterized by test sensitivity (${S}_{e}$) and specificity (${S}_{p}$). This is certainly the case with COVID-19 antibody tests developed during the first months of the pandemic. Therefore, there is a possibility that *X* includes some false positives and that some true positives are not counted in *X*.

We propose the following adjustment to Eqs. (1) and (2),

$$\begin{gathered} \alpha >P\left( {X<d|{p_u},{S_e},{S_p},n,N} \right) \\ =\sum\limits_{{i=0}}^{{d - 1}} {\sum\limits_{{k=0}}^{n} {\sum\limits_{{l=0}}^{k} {P\left( {T_{+}^{+}=l|{D^+}=k,{S_e}} \right)} } }\times \\ \quad P\left( {T_{ - }^{+}=i - l|{D^+}=k,{S_p}} \right)\times \\ \quad P\left( {{D^+}=k|N,n,{p_u}} \right) \\ \end{gathered}$$

$$\begin{gathered} \beta >P\left( {X\underline {>}d|{p_l},{S_e},{S_p},n,N} \right) \\ =\sum\limits_{{i=d}}^{n} {\sum\limits_{{k=0}}^{n} {\sum\limits_{{l=0}}^{k} {P\left( {T_{+}^{+}=l|{D^+}=k,{S_e}} \right)} } }\times \\ \quad P\left( {T_{ - }^{+}=i - l|{D^+}=k,{S_p}} \right)\times\\ \quad P\left( {{D^+}=k|N,n,{p_l}} \right) \\ \end{gathered}$$

where ${D}^{+}$ denotes the number of persons with the trait of interest chosen in the sample of size *n* and has a hypergeometric distribution with parameters *N*, *n*, and ${p}_{i}$ ($i$ corresponds to the lower or upper prevalence thresholds). Let ${T}_{+}^{+}$ denote the number of true positives given by a binomial distribution with parameters ${D}^{+}$ and ${S}_{e}$ and ${T}_{-}^{+}$ denote the number of false positives given by a binomial distribution with parameters $n-{D}^{+}$ and $1-{S}_{p}$. The proof is given in the online Supplementary Material 1.

We refer to this modified system as LQAS-IMP (Lot Quality Assurance Sampling for Imperfect tests). These adjustments are important to reflect the true errors of misclassification. Once they are made, $\alpha$ and $\beta$ will still account for uncertainty due to sampling (as is typical in LQAS), but will now also account for the uncertainty due to using imperfect tests. These modified equations reduce to Eqs. (1) and (2) under a perfect diagnostic test with sensitivity and specificity equal to one.

We make one cautionary note. It may be tempting to simply adjust the upper and lower prevalence thresholds to account for sensitivity and specificity of the test, that is substituting ${p}_{{i}^{*}}$ as defined below for ${p}_{i}$ in Eqs. (1) and (2),

$${p}_{{i}^{*}}= \left( {p}_{i} \times {S}_{e}\right)+\left(1-{p}_{i}\right)\times(1-{S}_{p})$$

This will result in invalid $\alpha$ and $\beta$ classification errors as this implicitly assumes that the sensitivity and specificity correspond to a fixed number of false positives and false negatives in a sample. Unlike the upper and lower prevalence thresholds, these probabilities cannot be considered fixed values for a given sample – for each individual tested, the probability of a false positive or false negative is drawn from a binomial distribution that depends on ${S}_{e}$and ${S}_{p}$ – whereas the underlying disease status is fixed for that individual at the outset.

## Results

### Application to serosurveillance in Haiti

At Partners In Health in Haiti, known locally as Zanmi Lasante (ZL), the goal was to assess the prior circulation of COVID-19 among healthcare workers (HCWs) at eleven facilities of varying sizes. HCWs have higher exposure risks than the general population, both in their clinical care and support activities and because they typically move more between home and facility locations. Therefore, HCWs can be monitored to detect any early evidence of disease circulation. If there is little-to-no evidence of COVID-19 antibodies in this high-risk population, then it is unlikely that SARS-CoV-2 is circulating or has circulated in the general population. Evidence of high antibody prevalence gives indication for the high-risk population’s exposure that can be followed with additional studies of population transmission dynamics.

Like other programs in LMICs, Zanmi Lasante has limited testing resources and needs to balance the resources used for this surveillance activity with other monitoring activities that require rapid antibody tests. For that reason, the team at Zanmi Lasante proposed designing a Lot Quality Assurance Sampling (LQAS) approach to classify facilities as having high or low COVID-19 antibody prevalence in HCWs. The available tests were antibody (IgG/IgM) rapid diagnostic tests. The ZL team originally estimated the sensitivity and specificity to be 90%, based on PIH synthesis of manufacturers details and other reports on test properties.

The Haiti team converged on a final set of parameters that all team members agreed met the overall program goals and aligned with their plans for next steps after the high and low classifications. Based on the Haiti team’s extensive knowledge of their health facilities and patient population, the parameter values chosen were ${p}_{u}=0.15$, ${p}_{l}=0.05$, and $\alpha =\beta =0.10$. That is, a “high prevalence” health facility is one where greater than or equal to 15% of workers test positive for antibodies and a “low prevalence” health facility is one where less than or equal to 5% of workers test positive for antibodies. The probability that a truly high prevalence health facility is mistakenly classified as low is 10%, and the probability that a truly low prevalence is mistakenly classified as high is 10%.

We note that the challenge with defining these parameters is that there is no inherently “right” or “wrong” value choice. Different programs will choose different values depending on their a priori knowledge of underlying prevalence levels and to overall goals and activities of that program. For instance, some programs may consider certain errors worse than others. Parameters may be set to either find more problem areas or, more often in the case of limited resources, set to only find the worst of the worst.

**Table 1:** LQAS-IMP systems account adjusting for antibody test specificity and sensitivity of 90% for eleven health facilities in Haiti (${p}_{u}=0.15$, ${p}_{l}=0.05$, and $\alpha =\beta =0.10$)

Health facility name	Population ***N***	Sample size ***n***	Decision rule ***d***
Hôpital Universitaire de Mirebalais	1373	149	27
Hôpital Bon Sauveur de Cange	655	144	26
Hôpital Saint Nicholas de Saint Marc	533	143	26
Hôpital Saint Thérèse de Hinche	228	121	22
Centre de Santé Charles Colimon de Petite Rivière de l’Artibonite	199	120	22
Centre de Santé de Lascahobas	184	109	20
Hôpital Dumarsais Estimé de Verettes	130	98	18
Centre de Santé de Thomonde	124	97	18
Centre de Santé de Cerca La Source	123	109	20
Hôpital la Nativité de Belladère^1^	110	108	20
Centre de Santé de Boucan Carre	108	98	18

^1^*n* and *d* values that minimized $\alpha$ and $\beta$ constraints were chosen

The sample sizes and decision rules for each health facility under our approach are given in Table 1. Importantly, for Hôpital la Nativité de Belladère, there were no values of *n* and *d* that satisfied the $\alpha$ and $\beta$ constraints, so we chose values that minimized both of these errors (see Simulation Study section). Under a perfect test (sensitivity and specificity both equal to one), the required sample size and decision rule would be *n* = 60 and *d* = 6 for Hôpital Universitaire de Mirebalais (*N* = 1373). As such, the required sample size more than doubles to account for diagnostic accuracy of the test. For prevalence estimation with a corresponding 95% confidence interval of length 10% (+/-5%), the sample size required would be *n* = 172 (assuming an underlying prevalence of 15%, perfect test accuracy, and N = 1373). Notably, the required sample size would be n = 251, a 68% increase in sample size, for prevalence estimate with a corresponding confidence interval of +/-5%, when accounting for an imperfect test sensitivity and specificity of 90%.

## Method performance and robustness to misspecified test properties: a simulation study

### Comparison of LQAS and LQAS-IMP for Haiti example

We validated our proposed approach and demonstrated the impact of adjusting for incorrect sensitivity and specificity of a test in a simulation study. To validate our approach, we calculated the $\alpha$ and $\beta$ classification errors under standard LQAS and LQAS-IMP for all health facilities in the Haiti example. Under standard LQAS, which implicitly assumes${S}_{e}= {S}_{p}= 1$, we calculated the sample size and decision rule for each health facility (Table 2). To simulate data for each health facility, we generated 3000 datasets of size *N* with (a) the true prevalence set at the probability threshold (${p}_{u}$ for $\alpha$; ${p}_{l}$ for $\beta$) to generate the true antibody status for a sampled individual and (b) test sensitivity and specificity of 90% to generate the observed test result for each person given their antibody status. We then computed $\alpha$ and $\beta$ using the sample sizes and decision rules from both Table 1 (adjusted) and Table 2 (standard) for comparison.

**Table 2:** LQAS systems assuming perfect antibody test accuracy for eleven health facilities in Haiti (${p}_{u}=0.15$, ${p}_{l}=0.05$, and $\alpha =\beta =0.10$)

Health facility name	Population ***N***	Sample size ***n***	Decision rule ***d***
Hôpital Universitaire de Mirebalais	1373	60	6
Hôpital Bon Sauveur de Cange	655	59	6
Hôpital Saint Nicholas de Saint Marc	533	59	6
Hôpital Saint Thérèse de Hinche	228	49	5
Centre de Santé Charles Colimon de Petite Rivière de l’Artibonite	199	48	5
Centre de Santé de Lascahobas	184	48	5
Hôpital Dumarsais Estimé de Verettes	130	39	4
Centre de Santé de Thomonde	124	39	4
Centre de Santé de Cerca La Source	123	40	4
Hôpital la Nativité de Belladère	110	47	5
Centre de Santé de Boucan Carre	108	39	4

**Fig. 1:**
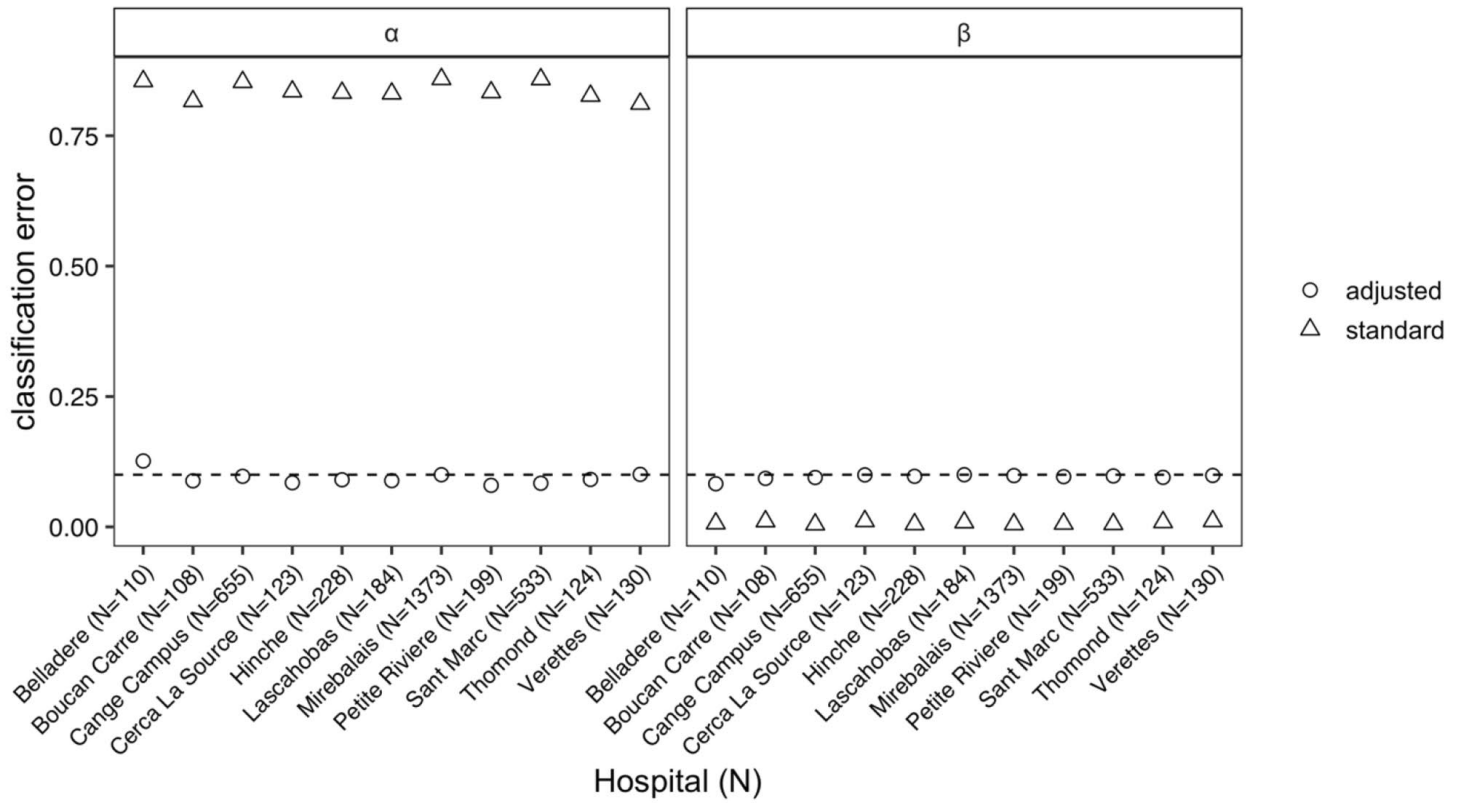
**Classification errors for each hospital from LQAS and LQAS-IMP.** Test sensitivity and specificity are equal to 90% and ${p}_{u}$=0.15 and ${p}_{l}$=0.05

Unsurprisingly, the standard LQAS required substantially smaller sample sizes than LQAS-IMP. Figure 1 shows the observed $\alpha$ and $\beta$ errors across the simulated datasets for each health facility. When the sensitivity and specificity are 90%, using the sample size and decision rule from the standard approach (that does not account for test properties) results in substantially higher $\alpha$ values than desired (ranging from 0.81 to 0.86 vs. 0.10). For example, the standard LQAS system resulted in 86% of the high antibody prevalence (${p}_{u}$=0.15) datasets for Hôpital Universitaire de Mirebalais being incorrectly classified as low prevalence. In contrast, the standard approach resulted in much lower $\beta$ values that specified (0.01 for all facilities vs. 0.10). The behavior of the classification errors are due to the low prevalence for *p*_*l*_ and *p*_*u*_, and if both were closer to one than we would observe overly high $\beta$ values and lower $\alpha$ values.

LQAS-IMP demonstrated $\alpha$ and $\beta$ values consistent with the targeted maximum classification error of 0.10. The one exception was for Hôpital la Nativité de Belladère, as our method could not find a system that satisfied the targeted classification error. The best system for this hospital resulted in $\alpha$=0.13 and $\beta$=0.08 as shown in Fig. 1.

### Robustness of classification errors to misspecified sensitivity and specificity in LQAS-IMP

We also varied the true test sensitivity and specificity from 0.85 to 0.95 to investigate changes in $\alpha$ and $\beta$ classification errors when the actual test sensitivity and specificity is different than what was specified in the LQAS-IMP design. We conducted the simulation for three upper and lower prevalence thresholds: low (${p}_{u}$=0.15 and ${p}_{l}$=0.05), medium (${p}_{u}$=0.55 and ${p}_{l}$=0.45), and high (${p}_{u}$=0.95 and ${p}_{l}$=0.85). For the three prevalence thresholds, we calculate the required *n* and *d* with LQAS-IMP accounting for sensitivity and specificity equal to 0.90 and $\alpha$ and $\beta$ error equal to 0.10, as in the Haiti setting. We computed the estimated $\alpha$ and $\beta$ from 3000 generated datasets of size *N* = 228 with a true prevalence set at the prevalence threshold (${p}_{u}$ for $\alpha$; ${p}_{l}$for $\beta$) and varied sensitivity and specificity. The LQAS-IMP system was *n* = 121 and *d* = 18 for the low probability threshold, *n* = 157 and *d* = 79 for the medium probability threshold, and *n* = 121 and *d* = 100 for the high probability threshold.

**Fig. 2:**
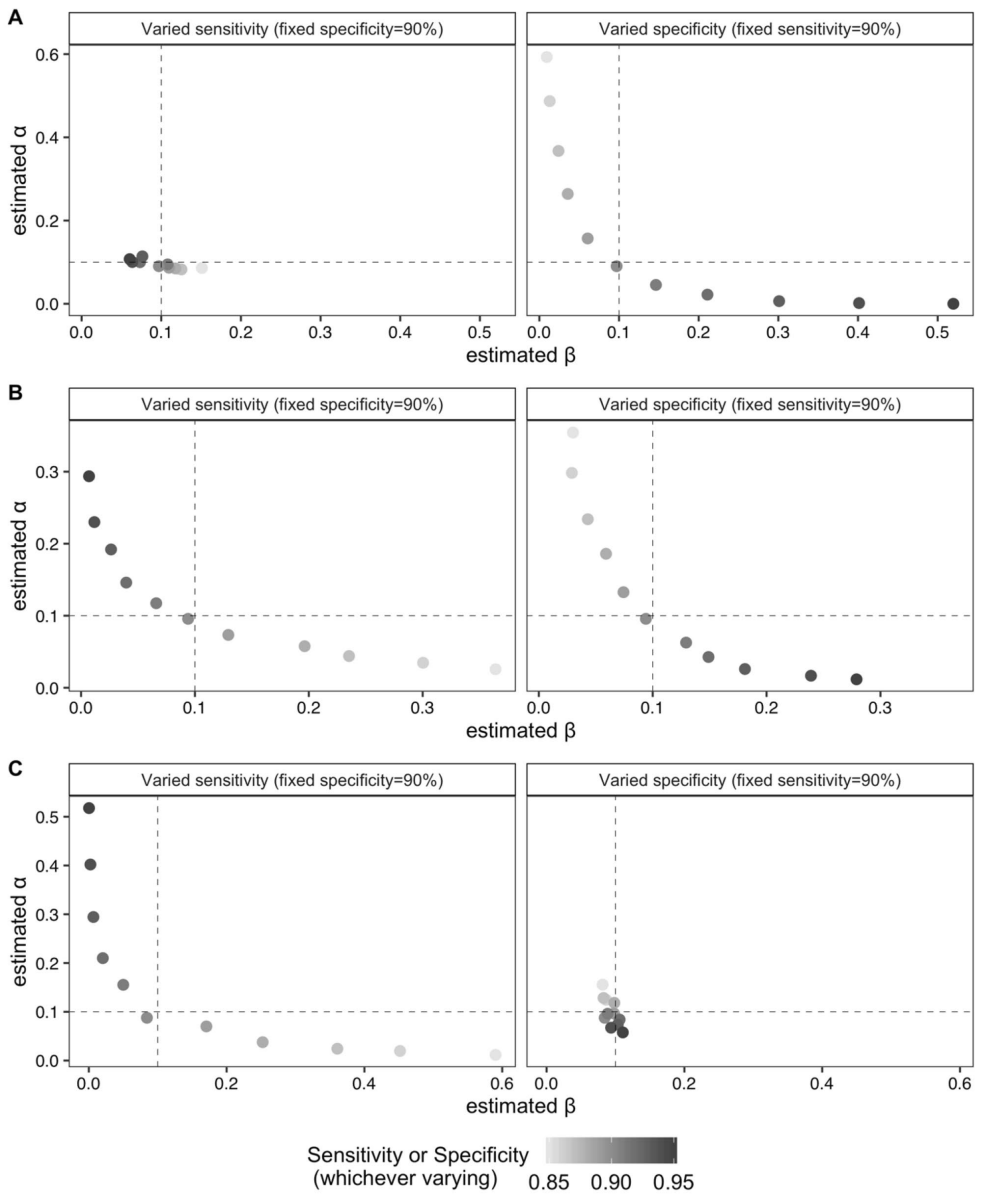
**Misclassification errors for varying values of true sensitivity and specificity under various prevalence thresholds.** The prevalence thresholds considered were (**A**) low, (**B**) medium, (**C**) high. The assumed sensitivity and specificity for adjustment was 90%. The dotted lines indicate pre-determined values of $\alpha$ and $\beta$. The black dot represents alignment between the true and assumed sensitivity and specificity values, which should always be contained in the lower left square

For the low prevalence threshold (Fig. 2 A), an incorrect assumed specificity greatly impacted the $\alpha$ and $\beta$ misclassification errors. That is, if one assumed the specificity was 90% in the LQAS-IMP design, but it was truly 89%, the $\alpha$ error would be 16% rather than the target 10%. However, in this scenario, an incorrect assumed sensitivity has minor impact on the classification errors. For the medium probability threshold (Fig. 2B), incorrect assumed specificity or sensitivity have major impacts on both classification errors. For the high probability threshold (Fig. 2 C), an incorrect assumed sensitivity impacts the $\alpha$ and $\beta$ misclassification errors, whereas an incorrect sensitivity has only minor impact.

## Discussion

### LQAS-IMP in the context of serosurveys

A requirement of the LQAS-IMP system is that test sensitivity and specificity are known and fixed quantities. Although estimates of sensitivity and specificity are often “known” as they are reported with diagnostic tests [10, 11], there may be variability in these estimates due to finite sample sizes in clinical agreement studies. For example, in the early months of COVID-19, the sample sizes for antibody test agreement studies was quite small (< 200 samples), resulting in wide confidence intervals for test sensitivity and specificity [12]. As shown in the simulation study, small deviations from the assumed sensitivity or specificity in LQAS-IMP can result in larger than expected misclassification probabilities. We encourage researchers to conduct similar simulation studies to assess the potential impact of incorrect sensitivity and specificity estimates on their systems. In the case of unknown or unreliable estimates of sensitivity and specificity, LQAS-IMP may not be suitable and prevalence estimation with appropriate adjustment is the best path forward [6, 7]. Ultimately, after evaluating the performance of LQAS-IMP and assessing our uncertainty of the COVID-19 antibody test properties, we decided that this approach was not appropriate for the Zanmi Lasante context. However, as these tests and our confidence in the test properties improve, this may change.

### Combining multiple LQAS-IMP procedures for prevalence estimation

As noted throughout, LQAS is a classification procedure. However, the results can also be used for estimation as the *n* individuals are a random sample [13]. Typically, estimation at the same level as classification is discouraged because the sample sizes are generally small resulting in confidence intervals that are too wide to be informative. In the Haiti case, there are multiple sites and combining samples across sites could produce overall estimates of antibody prevalence with high precision. The estimation process is straightforward; prevalence and variance estimates can be calculated from basic formulae from stratified sampling theory using standard software. However, the final prevalence and variance estimates must account for the imperfect sensitivity and specificity of the tests. These methods are well-defined [6], with recent extensions to COVID-19 prevalence estimates [7].

## Conclusion

The LQAS-IMP procedure allows teams to design assessments to extend the advantage of the positive features of LQAS to measures based on tests with imperfect properties. We originally designed this method to support Zanmi Lasante’s COVID-19 serosurveys. As we assessed the method’s properties, and specifically the vulnerability to misspecified sensitivity and specificity, we ultimately decided it was premature to deploy this method for this purpose. However, as COVID-19 antibody tests improve and the properties better quantified, LQAS-IMP may provide a rapid way to assess the state of COVID-19 burden in a context. This method can also be readily applied to monitoring other diseases.

## Electronic supplementary material

Below is the link to the electronic supplementary material.

Supplementary Material 1

## Data Availability

The code for LQAS-IMP is available at https://github.com/isabelfulcher/lqas_imp.

## List of abbreviations

LQAS: Lot Quality Assurance Sampling.
LMIC: Low- and Middle-Income Country.
ZL: Zanmi Lasante.
