# Supplementary material for: Adapting Lot Quality Assurance Sampling to accommodate imperfect diagnostic tests: application to COVID-19 serosurveillance in Haiti
Source: BMC Public Health. 2022 Nov 29;22:2221. doi: 10.1186/s12889-022-14206-5 (PMC9707425; doi:10.1186/s12889-022-14206-5)
Supplement: Supplementary file 1 — Supplementary Material 1 [file 12889_2022_14206_MOESM1_ESM.pdf]

Supplementary material: Adapting Lot Quality Assurance Sampling to accommodate imperfect tests: application to COVID-19 serosurveillance in Haiti

Derivation of LQAS-IMP system

$$\begin{aligned}
\beta &\geq P(X \geq d; p_l, S_e, S_p, n, N) \text{ (note: for ease of exposition, parameters are dropped)} \\
&= \sum_{i=d}^{n} P(X = i) \\
&= \sum_{i=d}^{n} \sum_{k=0}^{n} P(X = i | D^+ = k) P(D^+ = k) \\
&= \sum_{i=d}^{n} \sum_{k=0}^{n} P(T_+^+ + T_-^+ = i | D^+ = k) P(D^+ = k) \\
&= \sum_{i=d}^{n} \sum_{k=0}^{n} \sum_{l=0}^{k} P(T_+^+ = l, T_-^+ = i - l | D^+ = k) P(D^+ = k) \\
&= \sum_{i=d}^{n} \sum_{k=0}^{n} \sum_{l=0}^{k} P(T_+^+ = l | D^+ = k; S_e) P(T_-^+ = i - l | D^+ = k; S_p) P(D^+ = k; N, n, p_l)
\end{aligned}$$

Under same logic as β,

$$\begin{aligned}
\alpha &\geq P(X < d; p_u, S_e, S_p, n, N) \\
&= \sum_{i=0}^{d-1} \sum_{k=0}^{n} \sum_{l=0}^{k} P(T_+^+ = l | D^+ = k; S_e) P(T_-^+ = i - l | D^+ = k; S_p) P(D^+ = k; N, n, p_u)
\end{aligned}$$
